# Factors associated with patient activation among individuals with depression within racial/ethnic groups in the United States

**DOI:** 10.1016/j.pmedr.2023.102299

**Published:** 2023-06-25

**Authors:** M. Janelle Cambron-Mellott, Nate Way, Jacqueline Pesa, Muideen Adigun, H. Jean Wright II

**Affiliations:** a Cerner Enviza, an Oracle Company, 2800 Rock Creek Parkway, Kansas City, MO 64117, USA; b Janssen Scientific Affairs, LLC, 1125 Trenton Harbourton Road, Titusville, NJ 08560, USA; c Behavioral Health and Justice Division, Department of Behavioral Health and Intellectual disAbility Services, City of Philadelphia, 1601 Market Street, Five Penn Center, 7th Floor, Philadelphia, PA 19103, USA; d Temple University, Psychology Department, Weiss Hall, 6th Floor, 1701 N 13th St, Philadelphia, PA 19122, USA

**Keywords:** Depression, Depression severity, Patient activation, Race/ethnicity, Social determinants of health

## Abstract

Increasing patient activation may be vital for improving quality of care for individuals with depression. Among adults with depression who reside in the United States, we sought to examine the association of depression severity, race/ethnicity, and household income with patient activation and within identify factors associated with patient activation within race/ethnicity groups. Data from the 2020 US National Health and Wellness Survey, a cross-sectional, general population survey, were used to identify White, Black/African American, Asian, and Hispanic respondents with self-reported physician-diagnosed depression. Generalized linear models were used to identify factors associated with patient activation. Analyses included 8,216 respondents (mean age = 44 years, 68.0% female). Depression severity was negatively associated with patient activation (β = −0.29, *p* < 0.001). Patient activation was significantly higher in Black vs. White respondents (β = 1.50, *p* = 0.001) and in respondents with a household income of $25,000–$49,999 (β = 0.96, *p* = 0.015), $50,000–$99,000 (β = 0.88, *p* = 0.031), and ≥$100,000 (β = 1.78, *p* < 0.001) vs. <$25,000. Adjusted mean patient activation scores were highest among Black respondents (61.1), followed by Hispanic (60.2), White (59.6), and Asian (59.0) respondents. Neither race/ethnicity nor household income moderated the relationship between depression severity and patient activation; however, the factors most strongly associated with patient activation differed by race/ethnicity. These results indicate that the pathway to improving patient activation in individuals with depression may vary by race/ethnicity. Understanding factors associated with patient activation can help inform the design of interventions to increase patient activation in individuals with depression.

## Highlights

• Depression severity, race/ethnicity, income were associated with patient activation.
• Black individuals diagnosed with depression reported highest patient activation.
• Factors associated with patient activation differed by race/ethnicity.

## 1 Introduction

Depression is a leading cause of disability in the United States (US) (World Health Organization, 2020) and is associated with significant patient-centric, societal, and economic burden (Egede et al., 2016, Greenberg et al., 2015, Will et al., 2020, World Health Organization, 2021). Racial minorities are more likely to delay or fail to seek treatment for mental health, more likely to receive poorer quality care, and less likely to receive optimal treatments for depression (Center for Behavioral Health Statistics and Quality, 2021, McGuire and Miranda, 2008). Further, relative to White individuals, racial minorities report experiencing more severe depression (McGuire and Miranda, 2008, Vyas et al., 2020).

Patient activation, defined as the motivation, knowledge, skills, and confidence to make effective decisions in managing one’s own healthcare, is vital for improving quality of care in the US (Greene & Hibbard, 2012). Patients low in activation are generally passive recipients of care, whereas those higher in activation are more proactive and likely to engage in recommended behaviors (Greene & Hibbard, 2012). Patients with higher activation are more likely to have a usual source of care, more likely to get preventive care, less likely to delay getting care (Hibbard & Cunningham, 2008), and have better health outcomes (Greene & Hibbard, 2012). Several studies have found lower patient activation in Black/African American individuals than in White individuals (Eliacin et al., 2018, Gwynn et al., 2016, Hibbard et al., 2008, Holt et al., 2021), leading to consideration of whether targeting improvements in patient activation among racial minorities could lead to reducing disparities in health outcomes.

Prior research has shown that presence of depression symptoms and greater depression severity are associated with lower patient activation (Bos-Touwen et al., 2015, Corbett et al., 2021, Gleason et al., 2016, Hibbard et al., 2005, Magnezi et al., 2014, Sacks et al., 2014). In one study, higher severity of depression at baseline was associated with lower patient activation scores throughout a 12-month study period (Corbett et al., 2021). Conversely, in another study, higher patient activation at baseline was associated with lower depression severity one year later (Sacks et al., 2014).

In the few studies examining racial/ethnic differences in patient activation among individuals with depression, most have only included White and Black/African American patients (Eliacin et al., 2018, Hack et al., 2022) and/or clinical populations with relatively small sample sizes and a mixture of mental health conditions (Alegría et al., 2008, Eliacin et al., 2018, Hack et al., 2022). These studies lack generalizability to the wider population of adults with depression in the US. They also have not examined the relations between race/ethnicity, depression severity, and patient activation. Further, prior studies have not examined whether the factors predicting patient activation are influenced by race/ethnicity (Gleason et al., 2016, Greene and Hibbard, 2012). The objective of this study was to examine the relationship of race/ethnicity and depression severity with patient activation and identify factors associated with patient activation among individuals with depression. Further because higher income has been associated with higher patient activation (Oi, 2012) and lower depression severity (Califf et al., 2022), the role of household income in relation to depression severity and patient activation was examined.

## 2 Methods

### 2.1 Study design and data source

This retrospective study used data from the 2020 National Health and Wellness Survey (NHWS), a nationally-representative, self-reported, cross-sectional online survey of approximately 75,000 residents 18 years or older from the general adult population in the US. Respondents were recruited through an existing, general-purpose (not healthcare-specific) web-based consumer panel. Quota sampling was used to ensure that the NHWS sample was representative of the US population in terms of sex, age, and race. The NHWS was reviewed by the Pearl Institutional Review Board and granted exemption status (IRB no.: 20-KANT-219). All participants provided informed consent electronically, and this study was conducted in accordance with Declaration of Helsinki.

### 2.2 Study sample

Respondents were included in analyses if they self-reported experiencing depression in the past 12 months and a physician diagnosis of depression; were of Hispanic ethnicity or White, Black/African American, or Asian race; and completed the 9-item Patient Health Questionnaire (PHQ-9) and Patient Activation Measure (PAM). Respondents were excluded if they reported ever experiencing or being diagnosed with bipolar disorder or schizophrenia, or if they screened positive for bipolar disorder on the Mood Disorder Questionnaire (N. Williams, 2017).

### 2.3 Measures

Patient characteristics included age, sex, race/ethnicity, marital status, education, employment status, household income, health insurance, days exercising in past month, comorbidity burden as measured by the Charlson comorbidity index (CCI) (Quan et al., 2011), and current prescription for treatment of depression.

Depression severity was measured by the PHQ-9 (Kroenke et al., 2001). Higher scores (range, 0–27) represent more severe depression. Scores of 5, 10, 15, and 20 represent cutoffs for mild, moderate, moderately severe, and severe depression, respectively (Kroenke et al., 2001).

Patient activation was assessed with the 13-item PAM (Hibbard et al., 2005). Higher scores (range, 0–100) represent greater activation. Respondents were also grouped into four levels of activation, from low (1) to high (4) (Hibbard et al., 2005).

Health-related quality of life (HRQoL) measures included the 7-item Generalized Anxiety Disorder Assessment (GAD-7; higher scores [range, 0–21] represent greater anxiety severity) (Spitzer et al., 2006); Medical Outcomes Study 36-Item Short Form Survey Instrument Version 2 mental component summary (MCS) and physical component summary (PCS) scores (standardized to have a mean of 50 for the general population; higher scores [range, 0–100] indicate better HRQoL) (Ware et al., 2007); Short Form 6 Dimensions (SF-6D) utility score (higher scores [range, 0–1] indicate better HRQoL) (Hanmer, 2009); EQ-5D index (range, 0–1, 0 = health state equivalent to death and 1 = health state equivalent to perfect health), and EQ visual analog scale (VAS; range, 0–100, endpoints are labeled “best imaginable health state” and “worst imaginable health state”) of the 5-level EQ-5D (EQ-5D-5L) (Herdman et al., 2011).

Healthcare resource use included the number of traditional health care provider (HCP) visits, psychiatrist visits, psychologist/therapist visits, emergency room (ER) visits, and hospitalizations in the past 6 months.

The Work Productivity and Activity Impairment (WPAI) questionnaire measured absenteeism (the percentage of work time missed because of one's health in the past seven days), presenteeism (the percentage of impairment experienced while at work in the past seven days because of one's health), overall work productivity loss (an overall impairment estimate that is a combination of absenteeism and presenteeism), and activity impairment (the percentage of impairment in daily activities because of one's health in the past seven days) (Reilly et al., 1993). Only respondents who reported being employed provided data for absenteeism, presenteeism, and overall work impairment.

Direct costs were calculated by annualizing the number of healthcare provider visits, ER visits, and hospitalizations and then multiplying by unit costs for each type of visit using the 2018 Medical Expenditure Panel Survey (MEPS) (Agency for Healthcare Research and Quality, n.d.). For indirect costs, hourly rates from the US Bureau of Labor Statistics (U.S. Bureau of Labor Statistics, n.d.) were applied to the absenteeism and presenteeism estimates obtained from the WPAI using the human capital method (Onukwugha et al., 2016).

### 2.4 Statistical analyses

Analyses were conducted using SPSS Version 28 (IBM). Descriptive statistics were used to characterize respondent characteristics and study variables in aggregate and by depression severity.

Bivariate analyses were undertaken to compare study variables by 1) race/ethnicity and 2) within each racial/ethnic group by depression severity (PHQ-9 score: none/minimal [0–4], mild [5–9], moderate [10–14], moderately severe to severe [>15]) using chi-square tests for categorical variables and ANOVAs and independent sample t-tests for omnibus and pairwise comparisons of continuous variables, respectively. Pairwise comparisons were adjusted for multiplicity with a Bonferroni correction.

Generalized linear models (GLMs), specifying a normal distribution and an identity link function, were used to assess the covariate-adjusted relationship of depression severity (continuous PHQ-9 score), race/ethnicity, and income with patient activation. The GLM was first run with only the main effects and covariates in the model. Next, interaction terms for race/ethnicity, depression severity, income, depression severity were added to the GLM. Continuous variables were mean centered prior to including them in the models. Covariates incorporated constructs of theoretical importance as well as those identified as significantly different between groups in bivariate analyses and included: age, sex, marital status, education, health insurance status, days exercising in past month, CCI score, and current prescription use for depression. A sensitivity analysis examined the relationship between depression severity and race/ethnicity with patient activation, without education, health insurance, and household income included in the model. This was done to understand the impact of controlling for socioeconomic factors that are traditionally lower in disadvantaged communities.

GLMs were also used to identify the factors most strongly associated with patient activation for each race/ethnicity group. Regression coefficients (β), standard errors, and *p*-values were reported.

## 3 Results

**Fig. 1:**
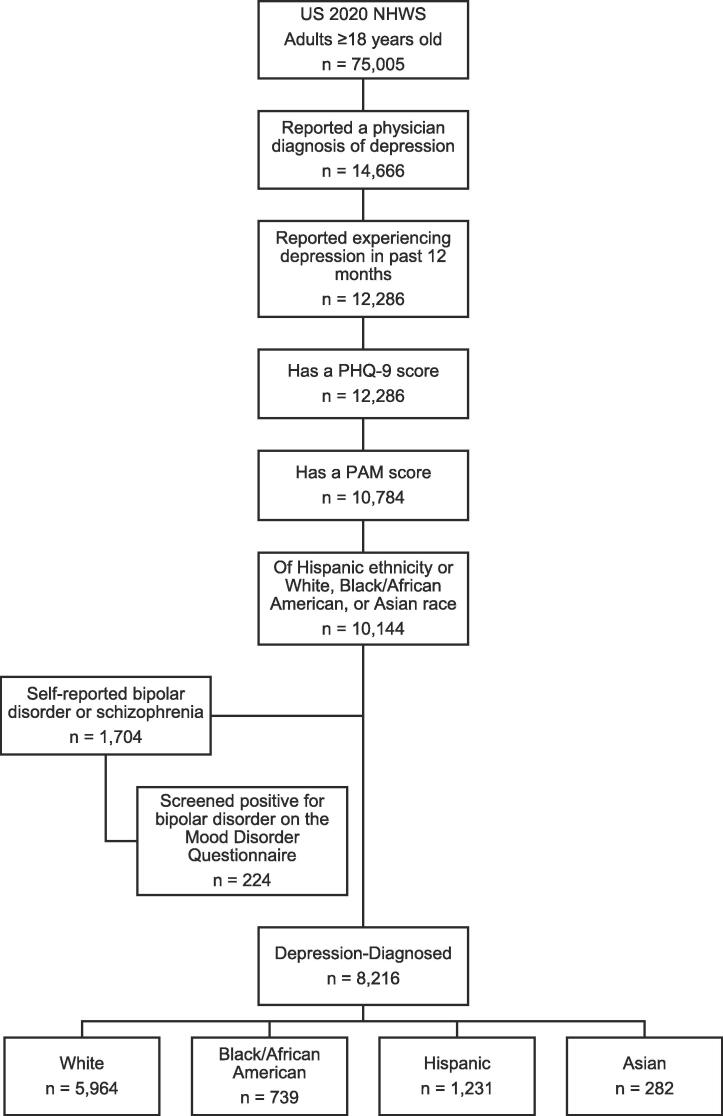
Study population. NHWS, National Health and Wellness Survey; PAM, Patient Activation Measure; PHQ-9, 9-item Patient Health Questionnaire.

In total, 8,216 respondents with self-reported and physician-diagnosed depression were included in the study (5,964 White, 739 Black, 1,231 Hispanic, 282 Asian; Fig. 1). The majority were female (68.0%); mean age was 43.97±15.94 years (Table 1). More than half were currently employed (55.1%) and on a prescription medication for depression (59.5%). The mean PAM score of the aggregate sample was 61.29±11.93.

**Table 1:** Sample characteristics, HRQoL, Work Productivity and Activity Impairment (WPAI), HCRU, and costs among adults with self-reported physician-diagnosed depression who participated in the 2020 US NHWS in aggregate and by depression severity.

	Total	Depression severity (PHQ-9 score)			
		Minimal (score 0–4)	Mild (score 5–9)	Moderate (score 10–14)	Moderately Severe/Severe (score 15–27)
	n = 8,216	n = 1,564	n = 2,670	n = 1,970	n = 2,012
Female, n (%)	5,587 (68.0)	980 (62.7)	1,830 (68.5)	1,401 (71.1)	1,376 (68.4)
Age, years, mean (SD)	43.97 (15.94)	49.29 (16.11)	45.44 (15.85)	41.49 (15.60)	40.32 (14.84)
Race/ethnicity					
White	5,964 (72.6)	1,241 (79.3)	1,981 (74.2)	1,392 (70.7)	1,350 (67.1)
Black/African American	739 (9.0)	132 (8.4)	219 (8.2)	177 (9.0)	211 (10.5)
Hispanic	1,231 (15.0)	150 (9.6)	368 (13.8)	337 (17.1)	376 (18.7)
Asian	282 (3.4)	41 (2.6)	102 (3.8)	64 (3.2)	75 (3.7)
Married/living with partner, n (%)	3,980 (48.4)	878 (56.1)	1,318 (49.4)	957 (48.6)	827 (41.1)
University degree or higher, n (%)	3,439 (41.9)	808 (51.7)	1,215 (45.5)	772 (39.2)	644 (32.0)
Employed, n (%)	4,525 (55.1)	891 (57.0)	1,495 (56.0)	1,120 (56.9)	1,019 (50.6)
Household income, n (%)					
<$25,000	1,641 (20.0)	211 (13.5)	474 (17.8)	391 (19.8)	565 (28.1)
$25,000 to <$50,000	1,968 (24.0)	338 (21.6)	626 (23.4)	494 (25.1)	510 (25.3)
$50,000 to <$100,000	2,674 (32.5)	532 (34.0)	912 (34.2)	637 (32.3)	593 (29.5)
≥$100,000	1,615 (19.7)	416 (26.6)	558 (20.9)	369 (18.7)	272 (13.5)
Decline to answer	318 (3.9)	67 (4.3)	100 (3.7)	79 (4.0)	72 (3.6)
Health insurance, n (%)					
Commercially insured	4,381 (53.3)	902 (57.7)	1,496 (56.0)	1,062 (53.9)	921 (45.8)
Medicaid	1,054 (12.8)	121 (7.7)	315 (11.8)	259 (13.1)	359 (17.8)
Medicare	1,574 (19.2)	381 (24.4)	523 (19.6)	327 (16.6)	343 (17.0)
Other type of insurance	385 (4.7)	63 (4.0)	125 (4.7)	92 (4.7)	105 (5.2)
Not insured	822 (10.0)	97 (6.2)	211 (7.9)	230 (11.7)	284 (14.1)
CCI score, mean (SD)	0.31 (0.94)	0.26 (0.84)	0.29 (0.96)	0.33 (0.89)	0.35 (1.00)
Days exercising, mean (SD)	6.82 (8.55)	8.26 (9.29)	7.33 (8.66)	6.18 (8.20)	5.63 (7.92)
Current prescription use for depression, yes, n (%)	4,885 (59.5)	995 (63.6)	1,554 (58.2)	1,162 (59.0)	1,174 (58.3)
PHQ-9 score, mean (SD)	10.25 (6.27)	2.36 (1.43)	7.03 (1.40)	11.86 (1.41)	19.08 (3.43)
PAM score, mean (SD)	61.29 (11.93)	64.69 (11.78)	62.35 (11.44)	59.94 (11.63)	58.56 (12.14)
PAM level, n (%)					
Level 1 (disengaged and overwhelmed)	810 (9.9)	65 (4.2)	156 (5.8)	248 (12.6)	341 (16.9)
Level 2 (becoming aware but still struggling)	1,570 (19.1)	211 (13.5)	476 (17.8)	437 (22.2)	446 (22.2)
Level 3 (taking action and gaining control)	4,228 (51.5)	856 (54.7)	1,487 (55.7)	959 (48.7)	926 (46.0)
Level 4 (maintaining behaviors and pushing further)	1,608 (19.6)	432 (27.6)	551 (20.6)	326 (16.5)	299 (14.9)

HRQoL					
GAD-7 score, mean (SD)	7.82 (5.41)	2.89 (3.09)	5.93 (3.63)	8.96 (4.27)	13.06 (4.95)
MCS score, mean (SD)	36.98 (11.16)	47.69 (8.00)	40.21 (8.25)	34.50 (8.19)	26.81 (9.50)
PCS score, mean (SD)	48.63 (10.56)	50.69 (9.02)	49.60 (10.11)	47.85 (10.76)	46.50 (11.55)
SF-6D utility score^3^	0.62 (0.11)	0.71 (0.10)	0.64 (0.09)	0.59 (0.08)	0.54 (0.09)
EQ-5D index score^4^	0.72 (0.16)	0.81 (0.11)	0.76 (0.12)	0.71 (0.14)	0.62 (0.17)
EQ VAS score	64.75 (22.98)	75.44 (19.09)	68.14 (21.12)	62.60 (22.01)	54.05 (24.16)

WPAI, mean (SD)					
Absenteeism (%)	11.13 (23.36)	4.43 (15.80)	7.68 (18.83)	13.23 (24.67)	19.80 (29.70)
Presenteeism (%)	29.89 (26.76)	14.76 (19.90)	24.58 (23.86)	35.78 (25.36)	45.29 (28.09)
Total work productivity impairment (%)	35.40 (31.31)	17.50 (24.07)	28.77 (27.90)	42.23 (29.91)	53.36 (31.68)
Activity impairment (%)	38.24 (28.48)	22.30 (24.40)	32.56 (26.38)	43.05 (26.51)	53.44 (27.28)

HCRU in past 6 months, mean (SD)					
Healthcare provider visits	5.56 (7.46)	4.34 (5.08)	5.22 (6.63)	5.86 (8.24)	6.65 (8.93)
Psychiatrist visits	0.44 (1.67)	0.30 (1.29)	0.34 (1.26)	0.46 (2.02)	0.67 (1.99)
Psychologist/therapist visits	1.34 (4.60)	0.71 (3.07)	1.16 (4.11)	1.54 (5.03)	1.89 (5.58)
ER visits	0.31 (1.02)	0.17 (0.55)	0.26 (0.93)	0.33 (1.18)	0.47 (1.20)
Hospitalizations	0.18 (1.29)	0.08 (0.41)	0.15 (1.02)	0.22 (2.15)	0.25 (0.85)

Annualized indirect costs ($), mean (SD)					
Absenteeism-related costs	4,052 (10,501)	1,623 (7,157)	2,733 (7,936)	4,741 (11,092)	7,366 (14,095)
Presenteeism-related costs	9,623 (11,901)	5,441 (8,528)	8,504 (11,155)	11,397 (12,015)	13,000 (13,888)
Total indirect costs	13,672 (16,371)	7,063 (11,803)	11,231 (14,396)	16,139 (16,554)	20,366 (19,133)

Annualized direct medical costs ($), mean (SD)					
HCP visits costs	3,481 (4,702)	2,706 (3,190)	3,265 (4,142)	3,666 (5,225)	4,190 (5,644)
ER visits costs	707 (2,350)	0,383 (1,203)	0,576 (2,146)	0,763 (2,722)	1,078 (2,798)
Hospitalizations costs	5,355 (36,616)	2,481 (12,075)	4,415 (29,273)	6,657 (60,568)	7,561 (24,906)
Total direct medical costs	9,543 (38,048)	5,570 (13,199)	8,256 (31,062)	11,086 (61,551)	12,829 (27,583)

ER, emergency room; GAD-7, 7-item general anxiety disorder scale; HCRU, healthcare resource use; HRQoL, health-related quality of life; MCS, mental component summary; NHWS, National Health and Wellness Survey; PAM, Patient Activation Measure, PCS, physical component summary; PHQ-9, 9-item Patient Health Questionnaire; WPAI, work productivity and activity impairment.

More than half of respondents with minimal depression were married/living with a partner (56.1%) and had a university degree or higher (51.7%). In contrast, less than half of respondents with mild, moderate, or moderately severe/severe depression were married/living with partner (49.4%, 48.6%, and 41.1%, respectively) and had a university degree or higher (45.5%, 39.2%, and 32.0%, respectively). More than half of respondents with minimal, mild, or moderate depression had a household income of ≥$50,000 (60.6%, 55.1%, and 51.1%, respectively) and were commercially insured (57.7%, 56.0%, and 53.9%, respectively). In contrast, less than half of those with moderately severe/severe depression had a household income ≥$50,000 (43.0%) and were commercially insured (45.8%) (Table 1).

### 3.1 Bivariate results: Sample characteristics and outcomes by race/ethnicity

White respondents were older than Black, Hispanic, and Asian respondents (45.93 [15.85] vs. 41.50 [15.39], 37.47 [14.51], and 37.45 [15.03] years, respectively, *p* < 0.001) (Table 2). Compared to Black, Hispanic, and Asian respondents, a higher proportion of White respondents were married/living with a partner (52.2% vs. 28.8%, 44.4%, and 38.7%, respectively, *p*s < 0.001) and taking a prescription medication for depression (62.1% vs. 54.9%, 50.9%, and 52.8%, respectively, *p*s < 0.05).

**Table 2:** Sample characteristics, HRQoL, Work Productivity and Activity Impairment (WPAI), HCRU, and costs by race/ethnicity among adults with self-reported physician-diagnosed depression who participated in the 2020 US NHWS.

	Race/Ethnicity				Omnibus *p*
	White	Black/African-American	Hispanic	Asian	
	n = 5,964	n = 739	n = 1,231	n = 282	
Female, n (%)	4025 (67.5)_a_	531 (71.9)_a_	843 (68.5)_a_	188 (66.7)_a_	0.106
Age, years, mean (SD)	45.93 (15.85)_a_	41.50 (15.39)_b_	37.47 (14.51)_c_	37.45 (15.03)_c_	<0.001
Married/living with partner, n (%)	3,112 (52.2)_a_	213 (28.8)_b_	546 (44.4)_c_	109 (38.7)_c_	<0.001
University degree or higher, n (%)	2,597 (43.5)_a_	251 (34.0)_b_	424 (34.4)_b_	167 (59.2)_c_	<0.001
Employed, n (%)	3,222 (54.0)_a_	394 (53.3)_a,b_	725 (58.9)_b,c_	184 (65.2)_c_	<0.001
Household income, n (%)					<0.001
<$25,000	1,071 (18.0)_a_	222 (30.0)_b_	295 (24.0)_c_	53 (18.8)_a,c_	
$25,000 to <$50,000	1,355 (22.7)_a_	219 (29.6)_b_	354 (28.8)_b_	40 (14.2)_c_	
$50,000 to <$100,000	2,022 (33.9)_a_	198 (26.8)_b_	366 (29.7)_b_	88 (31.2)_a,b_	
≥$100,000	1,288 (21.6)_a_	76 (10.3)_b_	168 (13.6)_b_	83 (29.4)_c_	
Decline to answer	228 (3.8)_a_	24 (3.2)_a_	48 (3.9)_a_	18 (6.4)_a_	
Health insurance, n (%)					<0.001
Commercially insured	3,265 (54.7)_a_	317 (42.9)_b_	611 (49.6)_c_	188 (66.7)_d_	
Medicaid	720 (12.1)_a_	124 (16.8)_b_	188 (15.3)_b_	22 (7.8)_a_	
Medicare	1,212 (20.3)_a_	166 (22.5)_a_	166 (13.5)_b_	30 (10.6)_b_	
Other type of insurance	254 (4.3)_a_	40 (5.4)_a,b_	76 (6.2)_b_	15 (5.3)_a,b_	
Not insured	513 (8.6)_a_	92 (12.4)_b_	190 (15.4)_b_	27 (9.6)_a,b_	
CCI score, mean (SD)	0.32 (0.97)_a_	0.32 (0.91)_a_	0.26 (0.85)_a,b_	0.15 (0.49)_b_	0.003
Days exercising, mean (SD)	6.86 (8.64)_a_	5.77 (7.75)_b_	6.84 (8.34)_a_	8.48 (9.32)_c_	<0.001
Current prescription use for depression, yes, n (%)	3,703 (62.1)_a_	406 (54.9)_b_	627 (50.9)_b_	149 (52.8)_b_	<0.001
PHQ-9 score, mean (SD)	9.91 (6.23)_a_	10.72 (6.51)_b_	11.49 (6.16)_c_	10.64 (6.33)_a,b,c_	<0.001
PHQ-9 depression severity, n (%)					<0.001
Minimal (0–*4)*	1,241 (20.8)_a_	132 (17.9)_a_	150 (12.2)_b_	41 (14.5)_a,b_	
Mild (5–*9)*	1,981 (33.2)_a_	219 (29.6)_a_	368 (29.9)_a_	102 (36.2)_a_	
Moderate (10–*14)*	1,392 (23.3)_a_	177 (24.0)_a,b_	337 (27.4)_b_	64 (22.7)_a,b_	
Moderately severe/severe (15–*27)*	1,350 (22.6)_a_	211 (28.6)_b_	376 (30.5)_b_	75 (26.6)_a,b_	

HRQoL					
GAD-7 score, mean (SD)	7.53_a_ (5.41)	8.29_b,c_ (5.54)	8.94_b_ (5.20)	7.96_a,c_ (5.29)	**<0.001**
MCS score, mean (SD)	37.49_a_ (11.28)	37.15_a,c_ (10.89)	34.73_b_ (10.51)	35.54_b,c_ (10.56)	**<0.001**
PCS score, mean (SD)	48.55_a_ (10.73)	47.23_b_ (10.37)	49.02_a_ (9.97)	52.18_c_ (8.95)	**<0.001**
SF-6D utility score^3^	0.62_a_ (0.11)	0.60_b_ (0.10)	0.60_b_ (0.10)	0.63_a_ (0.09)	**<0.001**
EQ-5D index score^4^	0.72_a_ (0.16)	0.72_a_ (0.16)	0.72_a_ (0.16)	0.76_b_ (0.14)	**0.001**
EQ VAS score	64.49_a_ (22.82)	64.65_a,b_ (24.00)	65.18_a,b_ (23.47)	68.63_b_ (21.08)	**0.026**

WPAI, mean (SD)					
Absenteeism (%)	9.57_a_ (21.79)	17.16_b_ (28.88)	15.06_b,c_ (26.67)	10.52_a,c_ (19.22)	**<0.001**
Presenteeism (%)	28.52_a_ (26.49)	32.93_b_ (28.00)	34.54_b_ (26.71)	29.64_a,b_ (26.59)	**<0.001**
Total work productivity impairment (%)	33.29_a_ (30.65)	41.58_b,c_ (33.99)	41.77_b_ (31.67)	34.39_a,c_ (30.60)	**<0.001**
Activity impairment (%)	37.65_a_ (28.51)	41.56_b_ (28.76)	40.24_b_ (28.19)	33.16_a_ (27.08)	**<0.001**

HCRU in past 6 months, mean (SD)					
Healthcare provider visits	5.61_a_ (7.40)	5.20_a_ (6.75)	5.57_a_ (7.99)	5.33_a_ (8.04)	0.532
Psychiatrist visits	0.41_a_ (1.47)	0.48_a,b_ (1.72)	0.57_b_ (2.38)	0.59_a,b_ (1.77)	**0.007**
Psychologist/therapist visits	1.31_a_ (4.58)	1.17_a_ (3.98)	1.46_a_ (4.59)	1.89_a_ (6.15)	0.108
ER visits	0.27_a_ (0.88)	0.45_b_ (1.30)	0.43_b_ (1.44)	0.20_a_ (0.63)	**<0.001**
Hospitalizations	0.14_a_ (0.60)	0.23_a,b_ (0.94)	0.35_b_ (2.96)	0.09_a_ (0.46)	**<0.001**

Annualized indirect costs ($), mean (SD)					
Absenteeism-related costs	3,551_a_ (9,705)	5,861_b_ (13,398	5,312_b_ (12,219)	4,130_a,b_ (8,932)	**<0.001**
Presenteeism-related costs	9,582_a_ (12,060)	9,286_a_ (12,095)	9,995_a_ (11,318)	9,601_a_ (10,946)	0.791
Total indirect costs	13,133_a_ (16,001)	15,116_a,b_ (17,494)	15,306_b_ (17,105)	13,708_a,b_ (16,950)	**0.003**

Annualized direct medical costs ($), mean (SD)					
HCP visits costs	3,516_a_ (4,680)	3,286_a_ (4,304)	3,475_a_ (4,987)	3,300_a_ (4,897)	0.569
ER visits costs	613_a_ (1,997)	1,061_b_ (3,041)	1,009_b_ (3,360)	458_a_ (1,469)	**<0.001**
Hospitalizations costs	4,275_a_ (18,299)	6,891_a,b_ (26,934)	10,294_b_ (82,602)	2,592_a_ (13,165)	**<0.001**
Total direct medical costs	8,403_a_ (20,725)	11,237_a,b_ (29,629)	14,779_b_ (83,501)	6,350_a_ (14,714)	**<0.001**

Note: Values in the same row and subtable not sharing the same subscript are significantly different at *p* < 0.05 in the two-sided test of equality for column proportions/means. Tests assume equal variances.

ER, emergency room; GAD-7, 7-item general anxiety disorder scale; HCRU, healthcare resource use; HRQoL, health-related quality of life; MCS, mental component summary; NHWS, National Health and Wellness Survey; PCS, physical component summary; PHQ-9, 9-item Patient Health Questionnaire; WPAI, work productivity and activity impairment.

Black and Hispanic respondents reported higher GAD-7 scores than White respondents (8.29 and 8.94 vs. 7.53, respectively, *p*s < 0.01) and lower SF-6D scores than White and Asian respondents (0.60 and 0.60 vs. 0.62 and 0.63, respectively, *p*s < 0.05). Hispanic and Asian respondents reported lower MCS scores than White respondents (34.73 and 35.54 vs. 37.49, respectively, *p*s < 0.05). Black and Hispanic respondents reported greater work productivity and activity impairment, more ER visits in the past 6 months, and greater indirect costs and ER costs, compared to White and Asian respondents (Table 2). Tables A1 – A4 show the bivariate comparisons of study variables for each race/ethnicity group by depression severity.

### 3.2 Bivariate results: Patient activation

Although PAM scores did not differ by race/ethnicity (Table 3), PAM levels varied as a function of race/ethnicity (*p* = 0.002); compared to White respondents, a higher proportion of Hispanic respondents were at Level 1 (12.8% vs. 9.1%, *p* < 0.001) and a lower proportion of Hispanic respondents were at Level 3 (47.5% vs. 52.4%, *p* = 0.012). Table A5 reports PAM by depression severity for each race/ethnicity group. Among White respondents, PAM scores were lower at higher levels of depression severity (64.76 minimal vs. 62.07 mild vs. 58.00 moderate vs. 58.22 severe, all pairwise comparisons *p* < 0.001). For Black, Hispanic, and Asian respondents, PAM scores were higher among those with minimal or mild depression compared to those with moderate or severe depression, although not all comparisons were statistically significant.

**Table 3:** Patient activation among depression-diagnosed adults who participated in the 2020 US NHWS by race/ethnicity.

	Race/Ethnicity				Omnibus *p*
	White	Black/African-American	Hispanic	Asian	
	n = 5,964	n = 739	n = 1,231	n = 282	
PAM score, mean (SD)	61.38_a_ (11.69)	61.81_a_ (12.44)	60.72_a_ (12.83)	60.48_a_ (11.30)	0.117

PAM level, n (%)					**0.002**
Level 1 (disengaged and overwhelmed)	545 (9.1)_a_	78 (10.6)_a,b_	158 (12.8)_b_	29 (10.3)_a,b_	
Level 2 (becoming aware but still struggling)	1,143 (19.2)_a_	133 (18.0)_a_	240 (19.5)_a_	54 (19.1)_a_	
Level 3 (taking action and gaining control)	3,123 (52.4)_a_	365 (49.4)_a,b_	585 (47.5)_b_	155 (55.0)_a,b_	
Level 4 (maintaining behaviors and pushing further)	1,153 (19.3)_a_	163 (22.1)_a_	248 (20.1)_a_	44 (15.6)_a_	

Note: Values in the same row and subtable not sharing the same subscript are significantly different at *p* < 0.05 in the two-sided test of equality for column proportions/means. Tests assume equal variances. NHWS, National Health and Wellness Survey; PAM; Patient Activation Measure.

### 3.3 Relationship of depression severity, race/ethnicity, and household income with patient activation

**Fig. 2:**
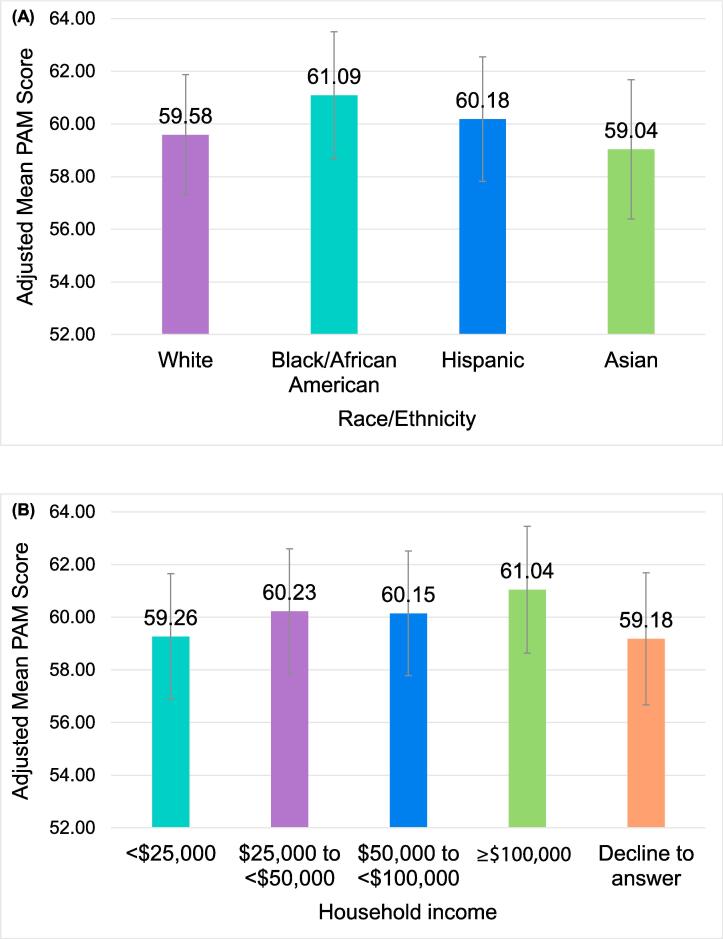
Adjusted mean PAM scores by a) race/ethnicity and b) household income among adults with self-reported physician-diagnosed depression who participated in the 2020 US NHWS Error bars represent 95% confidence intervals. NHWS, National Health and Wellness Survey; PAM, Patient Activation Measure.

After adjusting for covariates, depression severity was negatively associated with patient activation; (*W*(1) *=* 188.41, *p* < 0.001); for each unit increase in PHQ-9 score, PAM decreased by 0.29 points (95% CI −0.331 to −0.249). Race/ethnicity (*W*(3) *=* 13.44, *p* = 0.004) and household income (*W*(4) *=* 16.01, *p* = 0.003) were also significantly associated with patient activation; specifically, PAM scores were significantly higher in Black vs. White respondents, and in respondents with household income of ≥$25,000 vs. <$25,000 (Table 4). Adjusted mean PAM scores were highest among Black respondents (61.1), followed by Hispanic (60.2), White (59.6), and Asian (59.0) respondents (Fig. 2a). Adjusted mean PAM scores were highest among respondents with a household income of ≥$100,000 (61.0) and lowest among respondents with a household income <$25,000 (59.3) and those with undisclosed household income (59.2) (Fig. 2b). Neither race/ethnicity (*W*(3) *=* 5.49, *p* = 0.139) nor household income (*W*(4) *=* 0.41, *p* = 0.982) moderated the relationship between depression severity and patient activation. The sensitivity analysis yielded similar results (Table A6).

**Table 4:** Association of depression severity, race/ethnicity, and household income with patient activation among adults with self-reported physician-diagnosed depression who participated in the 2020 US NHWS: parameter estimates.

	β (SE)	*p*-value
Depression severity (PHQ-9 score)^1^	−0.290 (0.021)	**<0.001**

Race/ethnicity^2^		
White (reference)	0	
Black/African American	1.503 (0.455)	**0.001**
Hispanic	0.596 (0.369)	0.106
Asian	−0.549 (0.707)	0.438

Household income^3^		
<$25,000 (reference)	0	
$25,000 to <$50,000	0.965 (0.398)	**0.015**
$50,000 to <$100,000	0.880 (0.407)	**0.031**
≥$100,000	1.780 (0.485)	**<0.001**
Decline to answer	−0.087 (0.721)	0.904

Note: continuous predictors were centered due to multicollinearity when including interaction term; controlling for age, sex, marital status, education, health insurance status, days exercising, CCI score, and current prescription use for depression.

NHWS, National Health and Wellness Survey; PHQ-9, 9-item Patient Health Questionnaire; SE, standard error.

1 Interpretation: For each 1-point increase in PHQ-9 score, the PAM score changes by an average of <β>, keeping other predictors constant.

2 Interpretation: PAM scores change by an average of <β> for <Black/African American, Hispanic, or Asian respondents> compared to White respondents, keeping all other predictors constant.

3 Interpretation: PAM scores change by an average of <β> for <respondents with household incomes of $25,000 to <$50,000, $50,000 to <$100,000, $100,000, or who declined to answer> compared to respondents with income <$25,000, keeping all other predictors constant.

### 3.4 Factors associated with patient activation for each race/ethnicity group

The factors most strongly associated with PAM varied between the four race/ethnicity groups (Table 5 and Table A7). For White respondents, being female (β = 1.70, *p* < 0.001), having a household income above $100,000 (β = 1.40, *p* = 0.012), and self-reported current prescription for treatment of depression (β = −0.72, *p* = 0.019) were associated with higher activation. For Black respondents, undisclosed household income (β = −5.49, *p* = 0.035), being uninsured (β = −4.73, *p* = 0.002), younger age (β = 0.16, *p* < 0.001), and fewer days exercising (β = 0.16, *p* = 0.008) were associated with lower patient activation. Among Hispanic respondents, being uninsured (β = −2.72, *p* = 0.01); having insurance other than commercial, Medicare, or Medicaid (β = −5.69, *p* < 0.001); poorer physical functioning (β = 0.20, *p* < 0.001); and poorer mental functioning (β = 0.16, *p* = 0.001) were associated with lower activation. Among Asian respondents, being female (β = 3.50, *p* = 0.008), better mental functioning (β = 0.37, *p* < 0.001), and higher anxiety (β = 0.37, *p* = 0.019) were associated with higher activation.

**Table 5:** Parameter estimates for strongest predictors of patient activation for each race/ethnicity group among adults with self-reported physician-diagnosed depression who participated in the 2020 US NHWS.

	β^1^ (SE)	*p*-value
White respondents (n = 5,964)		
Sex: female (ref: male)	1.696	<0.001
Annual household income (ref: <$25,000)		
≥100,000	1.400	0.012
Current prescription for depression: no (ref: Yes)	−0.717	0.019

Black/African American respondents (n = 739)		
Annual household income (ref: <$25,000)		
Decline to answer	−5.491	0.035
Insurance status (ref: commercially insured)		
Not insured	−4.734	0.002
Age, years	0.157	<0.001
Days exercising in past month	0.156	0.008

Hispanic respondents (n = 1,231)		
Insurance status (ref: commercially insured)		
Other type of insurance^2^	−5.690	<0.001
Not insured	−2.723	0.010
PCS score	0.199	<0.001
MCS score	0.163	0.001

Asian respondents (n = 282)		
Sex: female (ref: male)	3.503	0.008
GAD-7 score	0.371	0.019
MCS score	0.370	<0.001

GAD-7, 7-item general anxiety disorder scale; HCP, healthcare provider; MCS, mental component summary; NHWS, National Health and Wellness Survey; PCS, physical component summary; ref, reference; SE, standard error.

1 Interpretation: continuous predictors – For each 1-unit increase in predictor, PAM score changes by an average of <β>, keeping other predictors constant; categorical predictors – PAM scores change by an average of <β> for <category> compared to reference group, keeping all other predictors constant.

2 Other type of insurance than commercial, Medicaid, or Medicare.

Some factors seemed to act as barriers toward patient activation for certain racial/ethnic groups, whereas those same factors acted as drivers toward patient activation for other racial/ethnic groups (Table 6). More healthcare provider visits were associated with greater patient activation among White and Hispanic respondents (β = 0.049 and β = 0.147, respectively) but with lower activation among Asian respondents (β = −0.247). Greater activity impairment was associated with higher patient activation among White and Asian respondents (β = −0.017 and β = 0.071, respectively) but with lower patient activation among Hispanic respondents (β = −0.040).

**Table 6:** Drivers and barriers of patient activation by race/ethnicity among adults with self-reported physician-diagnosed depression who participated in the 2020 US NHWS.

GAD-7, 7-item Generalized Anxiety Disorder assessment; HCP, healthcare provider; MCS, mental component summary; NHWS, National Health and Wellness Survey; PCS, physical component summary; PHQ-9, 9-item Patient Health Questionnaire.

Note: tables depict the predictors with a statistically significant association with patient activation by race/ethnicity; “+” = predictor showed an association with higher patient activation; “-” = predictor showed an association with lower patient activation; yellow highlights depict the strongest predictors for each race/ethnicity group.

^1^ Reference: Household income <$100,000.

^2^ Reference: Commercial insurance.

^3^ From Work Productivity and Activity Impairment questionnaire.

## 4 Discussion

This study provides novel insight into the relationship between race/ethnicity, depression severity, and patient activation in a large-scale representative sample of community-dwelling US adults with depression. In adjusted models, we found that patient activation was lower at higher levels of depression severity; the relationship between depression severity and patient activation did not differ as a function of race/ethnicity or household income. Instead, race/ethnicity and household income were independently associated with patient activation. Importantly, in adjusted models, patient activation was highest among Black respondents, followed by Hispanic, White, and Asian individuals. Further, drivers of higher patient activation differed by race/ethnicity, suggesting a potential role for race-specific tailored interventions seeking to increase activation and reduce the burden of depression.

### 4.1 Depression severity and patient activation

Our finding that depression severity is negatively associated with patient activation is consistent with prior research (Bos-Touwen et al., 2015, Corbett et al., 2021, Gleason et al., 2016, Sacks et al., 2014). This suggests that those most in need of help (i.e., severe depression) are the ones who are least activated. Therefore, among people with depression, interventions aiming to increase activation in populations where activation is low may be best served by targeting individuals with more severe depression.

### 4.2 Race/ethnicity and patient activation

Black respondents had significantly higher adjusted patient activation scores than White respondents. Although not statistically significant, Hispanic respondents also had higher adjusted patient activation scores than White respondents. These findings contradict prior research showing lower activation among minority groups in the US (Alexander et al., 2014, Cunningham et al., 2011, Hibbard et al., 2008, Smith et al., 2015), including among those receiving mental health care (Alegría et al., 2008, Eliacin et al., 2018, Hack et al., 2022). This discrepancy may be attributed to methodological differences between studies, including differences in populations studied, study designs, and statistical analyses utilized. For example, although our study utilized data from a nationally representative survey of the general population, sampling biases of other studies may have limited the generalizability of results (Alegría et al., 2008, Eliacin et al., 2018, Hack et al., 2022). Indeed, another study utilizing a US geographically representative sample of adults with chronic conditions found that Black patients were more activated than White patients (Imeri et al., 2023).

In this study we assessed the association between race/ethnicity and PAM in three ways: a) multivariable analysis with all covariates included in models to assess the “clean” association between race/ethnicity and PAM; b) a sensitivity analysis with factors that typically systematically differ by racial groups (income, education, and insurance) removed as covariates to partially isolate the association between race/ethnicity and PAM (these findings mirrored those of the first analysis); c) unadjusted bivariate analysis without covariates to allow for the closest approximation of how PAM levels fluctuate between racial/ethnic groups in the real world (these findings indicated virtually no differences in PAM by race/ethnicity). Collectively, these results indicate that there are not racial/ethnic groups in the US that are particularly low in PAM who are in most need of interventions (unadjusted analysis). Instead, results suggest that there may be aspects of race/ethnicity itself (i.e., perhaps aspects of racial/ethnic identity/culture) that push PAM levels in divergent directions for members of different races/ethnicities (adjusted analysis). Future research is certainly needed to understand the complex interrelationship of socioeconomic factors, race/ethnicity, and patient activation.

The diagnosis and treatment of mental health conditions remains heavily stigmatized in society and can represent a significant barrier to accessing effective care (Barney et al., 2006, Clement et al., 2015, Corrigan and Watson, 2002, Eylem et al., 2020, Korszun et al., 2012), particularly among minority communities who often face healthcare access issues (DeFreitas et al., 2018, Eylem et al., 2020). Stigma has empirically been associated with lower patient activation (Kato et al., 2016, Kato et al., 2020). Thus, those from minority communities (i.e., Black or Hispanic) experiencing depression may require even greater motivation to seek help and engage in treatment. Because our study included respondents self-reporting a diagnosis of depression, it may be that the relatively high levels of activation observed among Black respondents in adjusted analyses were a result of this patient population requiring greater activation to overcome barriers such as stigma and limited access to ultimately seek out a diagnosis.

In bivariate analyses, Hispanic and Black respondents reported worse health-related outcomes than their White and Asian peers, including greater work and activity impairment, more healthcare resource use, and poorer HRQoL. This yields an important and paradoxical finding: Black and Hispanic individuals possessed the highest levels of activation in adjusted comparisons, yet in unadjusted comparisons, they still experienced the poorest health outcomes. This suggests that although racial minorities may be able to overcome stigma and access barriers, inequities within the healthcare system trump the benefits of patient activation, as minorities tend to receive poorer quality mental health services compared to White individuals (US Department of Health and Human Services, 2001). This provides fertile ground for future research examining the complex relationship between race/ethnicity, patient activation, and care seeking in the context of depression.

### 4.3 Drivers and barriers of patient activation

We also found that drivers and barriers of patient activation varied by race/ethnicity. Drivers of patient activation included being female, having higher income, and using a prescription medication for depression among White respondents; better mental and physical health for Hispanic respondents; and being female, having higher anxiety, and having better mental health for Asian respondents. Barriers of patient activation included younger age, being uninsured, and being less physically active for Black respondents and being uninsured for Hispanic respondents.

High income was a driver among White individuals only. Though speculative, it may be the case that for minorities, the positive relationship between income and PAM is trumped by other factors that are negatively associated with PAM (e.g., education, insurance), which are more prominent in minority groups. In a similar manner, the fact that female gender was associated with greater activation for White and Asian respondents might also reflect different experiences in the healthcare system, with men tending to report more stigma than women around mental health disorders and thus seeking out care less often (Mackenzie et al., 2007, Pattyn et al., 2015). The fact that lack of insurance was identified as a barrier of patient activation for both Black and Hispanic individuals likely reflects the barrier that uninsured status plays in accessing healthcare for these individuals (Kaiser Family Foundation, 2022). Because such factors as gender, income, and insurance status are less amenable to change, these findings might instead be used to highlight certain members of groups who require greater attention (e.g., White and Asian men, uninsured Black and Hispanic individuals).

That physical activity emerged as a driver among Black respondents suggests that interventions could indirectly increase patient activation through encouraging more physical activity for Black participants. Indeed, previous research has reported an association between physical activity and health care seeking (Katz & Pronk, 2014), which the current study extends by identifying potential racial/ethnic differences in the context of depression. The fact that higher anxiety among Asian respondents was associated with higher patient activation may be suggestive that higher anxiety prompts care-seeking among this population. Future research is needed to understand the role of higher anxiety in patient activation. Overall, our findings suggest that the pathway to increasing patient activation in individuals with depression may vary by race/ethnicity, and thus tailored interventions may be needed to help address the well documented disparities in care among minority populations (McGuire and Miranda, 2008, Miranda and Cooper, 2004).

This study has limitations. The data collected were self-reported via survey without independent verification of the variables (e.g., diagnoses). Further, the cross-sectional nature of the study meant that causality could not be established between variables. As with any self-report survey, there can be potential bias from inaccurate recall or false reporting. Also, the web-based survey administration may have led to the under-representation of those that may not have had internet access or comfort with online interaction. Finally, some groups were represented by small sample sizes (e.g., Asian respondents with minimal depression) and thus the study may have been unable to detect small or medium effect sizes among such groups.

## 5 Conclusions

Results from the current study provide novel information about patient activation among individuals with depression and highlight potential differences that may exist in the factors that drive activation among various race/ethnicity groups. It is possible that the pathway to improving patient activation among those diagnosed with depression may vary by race/ethnicity. Thus, tailored interventions are needed to improve care and reduce the burden of this condition.

## CRediT authorship contribution statement

**M. Janelle Cambron-Mellott:** Conceptualization, Formal analysis, Investigation, Methodology, Project administration, Visualization, Writing – original draft, Writing – review & editing. **Nate Way:** Conceptualization, Formal analysis, Investigation, Methodology, Project administration, Supervision, Writing – original draft, Writing – review & editing. **Jacqueline Pesa:** Conceptualization, Methodology, Project administration, Resources, Supervision, Writing – review & editing. **Muideen Adigun:** Methodology, Project administration, Resources, Supervision, Writing – review & editing. **H. Jean Wright II:** Validation, Writing – review & editing.

## Declaration of Competing Interest

The authors declare the following financial interests/personal relationships which may be considered as potential competing interests: **M. J. Cambron-Mellott** and **N. Way** are employees of Cerner Enviza, which received funding from Janssen Scientific Affairs, LLC, to conduct and report on the study. **J. Pesa** and **M. Adigun** are employees of Janssen Scientific Affairs, LLC and stockholders of Johnson & Johnson. **H. J. Wright II** reported he had no disclosures and that he did not receive any honoraria/payments for this study.

## Data Availability

The data that have been used are confidential.
