# Supplementary material for: Factors associated with patient activation among individuals with depression within racial/ethnic groups in the United States
Source: Prev Med Rep. 2023 Jun 25;35:102299. doi: 10.1016/j.pmedr.2023.102299 (PMC10372381; doi:10.1016/j.pmedr.2023.102299)
Supplement: Supplementary data 1 [file mmc1.pdf]

Appendix A.

Table A1. Sample characteristics, HRQoL, Work Productivity and Activity Impairment (WPAI), HCRU, and costs by depression severity among White respondents with self-reported physician-diagnosed depression

	**Depression severity (PHQ-9 score)**				
	**Minimal (Score 0-4) n=1,241**	**Mild (Score 5-9) n=1,981**	**Moderate (Score 10-14) n=1,392**	**Moderately Severe/Severe (Score 15-27) n=1,350**	**Omnibus *p***
Female, n (%)	776 (62.5)$_{a}$	1,333 (67.3)$_{b}$	1,009 (72.5)$_{c}$	907 (67.2)$_{a,b}$	**<0.001**
Age, years, mean (SD)	50.35$_{a}$ (16.11)	47.27$_{b}$ (15.90)	43.38$_{c}$ (15.41)	42.55$_{c}$ (14.73)	**<0.001**
Married/living with partner, n (%)	742 (59.8)$_{a}$	1,034 (52.2)$_{b}$	729 (52.4)$_{b}$	607 (45.0)$_{c}$	**<0.001**
University degree or higher, n (%)	652 (52.5)$_{a}$	923 (46.6)$_{b}$	571 (41.0)$_{c}$	451 (33.4)$_{d}$	**<0.001**
Employed, n (%)	696 (56.1)$_{a}$	1,080 (54.5)$_{a}$	780 (56.0)$_{a}$	666 (49.3)$_{b}$	**<0.001**
Household income, n (%)					**<0.001**
<$25,000	155 (12.5)$_{a}$	306 (15.4)$_{a,b}$	252 (18.1)$_{b}$	358 (26.5)$_{c}$	
$25,000 to <$50,000	253 (20.4)$_{a}$	448 (22.6)$_{a}$	332 (23.9)$_{a}$	322 (23.9)$_{a}$	
$50,000 to <$100,000	426 (34.3)$_{a}$	698 (35.2)$_{a}$	463 (33.3)$_{a}$	435 (32.2)$_{a}$	
$100,000 +	354 (28.5)$_{a}$	451 (22.8)$_{b}$	289 (20.8)$_{b}$	194 (14.4)$_{c}$	
Decline to answer	53 (4.3)$_{a}$	78 (3.9)$_{a}$	56 (4.0)$_{a}$	41 (3.0)$_{a}$	
Health insurance, n (%)					**<0.001**
Commercially insured	718 (57.9)$_{a}$	1,122 (56.6)$_{a}$	788 (56.6)$_{a}$	637 (47.2)$_{b}$	
Medicaid	92 (7.4)$_{a}$	205 (10.3)$_{b}$	186 (13.4)$_{c}$	237 (17.6)$_{d}$	
Medicare	307 (24.7)$_{a}$	431 (21.8)$_{a,c}$	225 (16.2)$_{b}$	249 (18.4)$_{b,c}$	
Other type of insurance	48 (3.9)$_{a}$	86 (4.3)$_{a}$	55 (4.0)$_{a}$	65 (4.8)$_{a}$	
Not insured	76 (6.1)$_{a}$	137 (6.9)$_{a}$	138 (9.9)$_{b}$	162 (12.0)$_{b}$	
CCI score, mean (SD)	0.24$_{a}$ (0.80)	0.30$_{a,b}$ (1.03)	43.38$_{b}$ (15.41)	0.39$_{b,c}$ (1.05)	**<0.001**
Days exercising, mean (SD)	8.22$_{a}$ (9.40)	7.48$_{a}$ (8.74)	6.05$_{b}$ (8.19)	5.55$_{b}$ (7.92)	**<0.001**
Current prescription use for depression, yes, n (%)	818 (65.9)$_{a}$	1,202 (60.7)$_{b}$	866 (62.2)$_{a,b}$	817 (60.5)$_{b}$	**0.013**
PHQ-9 score, mean (SD)	2.38$_{a}$ (1.43)	7.01$_{b}$ (1.40)	11.85$_{c}$ (1.40)	19.11$_{d}$ (3.42)	**<0.001**
HRQoL					
GAD-7 score, mean (SD)	2.86$_{a}$ (3.01)	5.75$_{b}$ (3.60)	8.86$_{c}$ (4.33)	13.07$_{d}$ (5.02)	**<0.001**
MCS score, mean (SD)	47.90$_{a}$ (7.86)	40.66$_{b}$ (8.18)	34.46$_{c}$ (8.35)	26.41$_{d}$ (9.44)	**<0.001**
PCS score, mean (SD)	50.72$_{a}$ (9.17)	49.55$_{b}$ (10.13)	47.59$_{c}$ (11.15)	46.09$_{d}$ (11.82)	**<0.001**

	Depression severity (PHQ-9 score)				
	**Minimal (Score 0-4) n=1,241**	**Mild (Score 5-9) n=1,981**	**Moderate (Score 10-14) n=1,392**	**Moderately Severe/Severe (Score 15-27) n=1,350**	**Omnibus *p***
SF-6D utility score[3]	0.71$_a$ (0.10)	0.65$_b$ (0.09)	0.59$_c$ (0.08)	0.53$_d$ (0.09)	**<0.001**
EQ-5D index score[4]	0.81$_a$ (0.11)	0.76$_b$ (0.12)	0.70$_c$ (0.15)	0.61$_d$ (0.17)	**<0.001**
EQ VAS score	75.40$_a$ (18.73)	68.38$_b$ (20.44)	61.06$_c$ (22.13)	52.30$_d$ (23.84)	**<0.001**
WPAI, mean (SD)					
Absenteeism (%)	3.62$_a$ (14.03)	5.89$_a$ (16.21)	11.81$_b$ (23.71)	19.11$_c$ (29.19)	**<0.001**
Presenteeism (%)	14.20$_a$ (19.27)	22.65$_b$ (22.82)	35.13$_c$ (25.30)	46.31$_d$ (28.27)	**<0.001**
Total work productivity impairment (%)	16.37$_a$ (22.70)	25.84$_b$ (26.17)	41.08$_c$ (29.51)	53.92$_d$ (31.51)	**<0.001**
Activity impairment (%)	21.77$_a$ (24.22)	31.73$_b$ (26.03)	43.54$_c$ (26.50)	54.86$_d$ (26.81)	**<0.001**
HCRU in past 6 months, mean (SD)					
Healthcare provider visits	4.37$_a$ (5.11)	5.34$_b$ (6.71)	6.04$_c$ (8.40)	6.69$_c$ (8.74)	**<0.001**
Psychiatrist visits	0.27$_a$ (1.25)	0.34$_a$ (1.30)	0.40$_a$ (1.39)	0.63$_b$ (1.89)	**<0.001**
Psychologist/therapist visits	0.71$_a$ (3.10)	1.15$_b$ (4.14)	1.55$_{b,c}$ (5.10)	1.86$_c$ (5.60)	**<0.001**
ER visits	0.16$_a$ (0.55)	0.24$_{a,b}$ (0.93)	0.29$_b$ (0.86)	0.40$_c$ (1.02)	**<0.001**
Hospitalizations	0.08$_a$ (0.40)	0.12$_{a,b}$ (0.54)	0.14$_b$ (0.63)	0.22$_c$ (0.78)	**<0.001**
Annualized indirect costs ($), mean (SD)					
Absenteeism-related costs	1,293$_a$ (6,246)	2,149$_a$ (7,123)	4,326$_b$ (10,360)	7,291$_c$ (13,583)	**<0.001**
Presenteeism-related costs	5,174$_a$ (8,072)	8,182$_b$ (10,831)	11,725$_c$ (12,357)	13,968$_d$ (14,807)	**<0.001**
Total indirect costs	6,467$_a$ (10,854)	10,331$_b$ (13,738)	16,051$_c$ (16,064)	21,260$_d$ (19,330)	**<0.001**
Annualized direct medical costs ($), mean (SD)					
HCP visits costs	2,713$_a$ (3,194)	3,339$_b$ (4,198)	3,793$_c$ (5,343)	4,225$_c$ (5,559)	**<0.001**
ER visits costs	352$_a$ (1,153)	528$_{a,b}$ (2,127)	658$_b$ (1,969)	928$_c$ (2,364)	**<0.001**
Hospitalizations costs	2,391$_a$ (11,775)	3,640$_{a,b}$ (16,302)	4,471$_b$ (20,406)	6,739$_c$ (22,876)	**<0.001**
Total direct medical costs ($)	5,456$_a$ (12,902)	7,507$_b$ (18,818)	8,923$_b$ (23,227)	11,892$_c$ (25,454)	**<0.001**

Note: Values in the same row and subtable not sharing the same subscript are significantly different at $p < .05$ in the two-sided test of equality for column proportions/means. Tests assume equal variances.

ER, emergency room; GAD-7, 7-item general anxiety disorder scale; HCRU, healthcare resource use; HRQoL, health-related quality of life; MCS, mental component summary; PCS, physical component summary; PHQ-9, 9-item Patient Health Questionnaire; WPAI, work productivity and activity impairment

Table A2. Sample characteristics, HRQoL, Work Productivity and Activity Impairment (WPAI), HCRU, and costs by depression severity among Black/African American respondents with self-reported physician-diagnosed depression

	**Depression severity (PHQ-9 score)**				
	**Minimal (Score 0-4) n=132**	**Mild (Score 5-9) n=219**	**Moderate (Score 10-14) n=177**	**Moderately Severe/Severe (Score 15-27) n=211**	**Omnibus *p***
Female, n (%)	94 (71.2)$_a$	161 (73.5)$_a$	122 (68.9)$_a$	154 (73.0)$_a$	0.750
Age, years, mean (SD)	47.42$_a$ (15.01)	42.97$_b$ (14.70)	38.50$_c$ (15.27)	38.78$_c$ (15.27)	**<0.001**
Married/living with partner, n (%)	46 (34.8)$_a$	67 (30.6)$_a$	45 (25.4)$_a$	55 (26.1)$_a$	0.386
University degree or higher, n (%)	61 (46.2)$_a$	92 (42.0)$_{a,b}$	53 (29.9)$_{b,c}$	45 (21.3)$_c$	**<0.001**
Employed, n (%)	71 (53.8)$_a$	122 (55.8)$_a$	101 (57.1)$_a$	100 (47.3)$_a$	0.213
Household income, n (%)					0.141
<$25,000	32 (24.2)$_a$	62 (28.3)$_a$	51 (28.8)$_a$	77 (36.5)$_a$	
$25,000 to <$50,000	41 (31.1)$_a$	56 (25.6)$_a$	57 (32.2)$_a$	65 (30.8)$_a$	
$50,000 to <$100,000	42 (31.8)$_a$	68 (31.1)$_a$	42 (23.7)$_a$	46 (21.8)$_a$	
$100,000 +	13 (9.8)$_a$	27 (12.3)$_a$	22 (12.4)$_a$	14 (6.6)$_a$	
Decline to answer	4 (3.0)$_a$	6 (2.7)$_a$	5 (2.8)$_a$	9 (4.3)$_a$	
Health insurance, n (%)					**<0.001**
Commercially insured	66 (50.0)$_a$	103 (47.0)$_a$	76 (42.9)$_{a,b}$	72 (34.1)$_b$	
Medicaid	12 (9.1)$_a$	42 (19.2)$_{a,b}$	26 (14.7)$_{a,b}$	44 (20.9)$_b$	
Medicare	41 (31.1)$_a$	43 (19.6)$_a$	39 (22.0)$_a$	43 (20.4)$_a$	
Other type of insurance	6 (4.5)$_a$	15 (6.8)$_a$	9 (5.1)$_a$	10 (4.7)$_a$	
Not insured	7 (5.3)$_a$	16 (7.3)$_{a,b}$	27 (15.3)$_{b,c}$	42 (19.9)$_c$	
CCI score, mean (SD)	0.42$_a$ (1.08)	0.34$_a$ (0.89)	0.23$_a$ (0.74)	0.32$_a$ (0.96)	0.321
Days exercising, mean (SD)	7.20$_a$ (8.48)	5.88$_{a,b}$ (7.56)	5.55$_{a,b}$ (7.60)	4.93$_b$ (7.51)	0.067
Current prescription use for depression, yes, n (%)	73 (55.3)$_a$	118 (53.9)$_a$	95 (53.7)$_a$	120 (56.9)$_a$	0.910
PHQ-9 score, mean (SD)	2.14$_a$ (1.48)	6.94$_b$ (1.37)	11.85$_c$ (1.43)	19.06$_d$ (3.43)	**<0.001**
HRQoL					
GAD-7 score, mean (SD)	2.65$_a$ (3.53)	6.34$_b$ (3.67)	9.02$_c$ (4.17)	13.23$_d$ (4.74)	**<0.001**
MCS score, mean (SD)	48.48$_a$ (7.87)	39.68$_b$ (8.37)	35.42$_c$ (8.00)	28.90$_d$ (9.68)	**<0.001**
PCS score, mean (SD)	48.76$_a$ (9.47)	47.69$_{a,b}$ (10.19)	47.56$_{a,b}$ (10.05)	45.52$_b$ (11.19)	**0.026**

	Depression severity (PHQ-9 score)				
	**Minimal (Score 0-4) n=132**	**Mild (Score 5-9) n=219**	**Moderate (Score 10-14) n=177**	**Moderately Severe/Severe (Score 15-27) n=211**	**Omnibus *p***
SF-6D utility score[3]	0.69$_{a}$ (0.11)	0.62$_{b}$ (0.08)	0.59$_{c}$ (0.09)	0.54$_{d}$ (0.10)	**<0.001**
EQ-5D index score[4]	0.81$_{a}$ (0.12)	0.75$_{b}$ (0.13)	0.71$_{b}$ (0.14)	0.62$_{c}$ (0.18)	**<0.001**
EQ VAS score	74.52$_{a}$ (20.35)	66.37$_{b}$ (22.83)	66.45$_{b}$ (22.08)	55.19$_{c}$ (25.68)	**<0.001**
WPAI, mean (SD)					
Absenteeism (%)	5.84$_{a}$ (18.43)	15.36$_{a,b}$ (27.26)	18.42$_{b,c}$ (26.57)	26.19$_{c}$ (35.42)	**<0.001**
Presenteeism (%)	15.85$_{a}$ (22.97)	30.87$_{b}$ (27.06)	37.82$_{b,c}$ (24.80)	43.53$_{c}$ (29.55)	**<0.001**
Total work productivity impairment (%)	19.93$_{a}$ (28.02)	39.42$_{b}$ (32.55)	46.07$_{b,c}$ (30.79)	55.53$_{c}$ (34.54)	**<0.001**
Activity impairment (%)	26.97$_{a}$ (27.68)	37.99$_{b}$ (28.41)	42.03$_{b}$ (25.17)	53.98$_{c}$ (27.54)	**<0.001**
HCRU in past 6 months, mean (SD)					
Healthcare provider visits	4.31$_{a}$ (4.69)	5.45$_{a}$ (5.53)	4.90$_{a}$ (7.16)	5.76$_{a}$ (8.41)	0.222
Psychiatrist visits	0.36$_{a}$ (1.59)	0.39$_{a}$ (1.18)	0.47$_{a}$ (1.35)	0.68$_{a}$ (2.40)	0.250
Psychologist/therapist visits	0.55$_{a}$ (2.49)	1.23$_{a}$ (3.19)	1.52$_{a}$ (4.92)	1.22$_{a}$ (4.53)	0.195
ER visits	0.26$_{a}$ (0.72)	0.38$_{a,b}$ (1.28)	0.42$_{a,b}$ (1.13)	0.68$_{b}$ (1.65)	**0.018**
Hospitalizations	0.16$_{a}$ (0.63)	0.19$_{a}$ (1.15)	0.25$_{a}$ (0.91)	0.29$_{a}$ (0.87)	0.538
Annualized indirect costs ($), mean (SD)					
Absenteeism-related costs	3,017$_{a}$ (11,855)	5,634$_{a}$ (12,018)	5,471$_{a}$ (13,097)	8,546$_{a}$ (15,797)	0.065
Presenteeism-related costs	6,422$_{a}$ (10,970)	9,449$_{a}$ (13,519)	9,788$_{a}$ (10,499)	10,641$_{a}$ (12,319)	0.148
Total indirect costs	9,439$_{a}$ (15,860)	14,990$_{a,b}$ (17,510)	15,259$_{a,b}$ (17,358)	19,187$_{b}$ (17,838)	**0.005**
Annualized direct medical costs ($), mean (SD)					
HCP visits costs	2,694$_{a}$ (2,948)	3,453$_{a}$ (3,543)	3,099$_{a}$ (4,639)	3,639$_{a}$ (5,310)	0.206
ER visits costs	602$_{a}$ (1,682)	890$_{a,b}$ (2,975)	988$_{a,b}$ (2,656)	1,587$_{b}$ (3,897)	**0.017**
Hospitalizations costs	4,891$_{a}$ (18,678)	5,651$_{a}$ (32,144)	7,157$_{a}$ (25,761)	9,205$_{a}$ (26,334)	0.430
Total direct medical costs ($)	8,187$_{a}$ (19,592)	9,995$_{a}$ (35,332)	11,244$_{a}$ (27,679)	14,430$_{a}$ (29,834)	0.237

Note: Values in the same row and subtable not sharing the same subscript are significantly different at $p < .05$ in the two-sided test of equality for column proportions/means. Tests assume equal variances.

ER, emergency room; GAD-7, 7-item general anxiety disorder scale; HCRU, healthcare resource use; HRQoL, health-related quality of life; MCS, mental component summary; PCS, physical component summary; PHQ-9, 9-item Patient Health Questionnaire; WPAI, work productivity and activity impairment

Table A3. Sample characteristics, HRQoL, Work Productivity and Activity Impairment (WPAI), HCRU, and costs by depression severity among Hispanic respondents with self-reported physician-diagnosed depression

	**Depression severity (PHQ-9 score)**				
	**Minimal (Score 0-4) n=150**	**Mild (Score 5-9) n=368**	**Moderate (Score 10-14) n=337**	**Moderately Severe/Severe (Score 15-27) n=376**	**Omnibus *p***
Female, n (%)	83 (55.3)$_{a}$	263 (71.5)$_{b}$	229 (68.0)$_{b}$	268 (71.3)$_{b}$	**0.002**
Age, years, mean (SD)	42.94$_{a}$ (15.82)	39.23$_{b}$ (14.01)	36.66$_{b,c}$ (15.12)	34.29$_{c}$ (12.97)	**<0.001**
Married/living with partner, n (%)	69 (46.0)$_{a}$	172 (46.7)$_{a}$	160 (47.5)$_{a}$	145 (38.6)$_{a}$	0.194
University degree or higher, n (%)	66 (44.0)$_{a}$	138 (37.5)$_{a}$	115 (34.1)$_{a,b}$	105 (27.9)$_{b}$	**0.010**
Employed, n (%)	94 (62.7)$_{a}$	221 (60.1)$_{a}$	202 (59.9)$_{a}$	208 (55.3)$_{a}$	0.358
Household income, n (%)					**0.004**
<$25,000	19 (12.7)$_{a}$	88 (23.9)$_{b}$	74 (22.0)$_{a,b}$	114 (30.3)$_{b}$	
$25,000 to <$50,000	41 (27.3)$_{a}$	107 (29.1)$_{a}$	98 (29.1)$_{a}$	108 (28.7)$_{a}$	
$50,000 to <$100,000	51 (34.0)$_{a}$	111 (30.2)$_{a}$	112 (33.2)$_{a}$	92 (24.5)$_{a}$	
$100,000 +	31 (20.7)$_{a}$	50 (13.6)$_{a}$	41 (12.2)$_{a}$	46 (12.2)$_{a}$	
Decline to answer	8 (5.3)$_{a}$	12 (3.3)$_{a}$	12 (3.6)$_{a}$	16 (4.3)$_{a}$	
Health insurance, n (%)					**0.006**
Commercially insured	84 (56.0)$_{a}$	199 (54.1)$_{a}$	161 (47.8)$_{a}$	167 (44.4)$_{a}$	
Medicaid	16 (10.7)$_{a}$	58 (15.8)$_{a}$	42 (12.5)$_{a}$	72 (19.1)$_{a}$	
Medicare	28 (18.7)$_{a}$	40 (10.9)$_{a}$	54 (16.0)$_{a}$	44 (11.7)$_{a}$	
Other type of insurance	8 (5.3)$_{a}$	20 (5.4)$_{a}$	24 (7.1)$_{a}$	24 (6.4)$_{a}$	
Not insured	14 (9.3)$_{a}$	51 (13.9)$_{a}$	56 (16.6)$_{a}$	69 (18.4)$_{a}$	
CCI score, mean (SD)	0.29$_{a}$ (0.95)	0.21$_{a}$ (0.66)	0.26$_{a}$ (0.90)	0.29$_{a}$ (0.93)	0.616
Days exercising, mean (SD)	8.83$_{a}$ (8.96)	7.05$_{a,b}$ (8.41)	6.72$_{a,b}$ (8.40)	5.94$_{b}$ (7.82)	**0.004**
Current prescription use for depression, yes, n (%)	79 (52.7)$_{a}$	185 (50.3)$_{a}$	167 (49.6)$_{a}$	196 (52.1)$_{a}$	0.869
PHQ-9 score, mean (SD)	2.35$_{a}$ (1.44)	7.20$_{b}$ (1.42)	11.88$_{c}$ (1.42)	19.00$_{d}$ (3.40)	**<0.001**
HRQoL					
GAD-7 score, mean (SD)	3.53$_{a}$ (3.46)	6.51$_{b}$ (3.54)	9.41$_{c}$ (4.13)	13.07$_{d}$ (4.66)	**<0.001**
MCS score, mean (SD)	45.40$_{a}$ (8.90)	38.77$_{b}$ (8.32)	34.15$_{c}$ (7.75)	27.05$_{d}$ (9.38)	**<0.001**
PCS score, mean (SD)	51.58$_{a}$ (7.41)	49.99$_{a,b}$ (9.99)	48.37$_{b,c}$ (9.56)	47.65$_{c}$ (10.89)	**<0.001**
SF-6D utility score[3]	0.69$_{a}$ (0.11)	0.63$_{b}$ (0.09)	0.58$_{c}$ (0.08)	0.55$_{d}$ (0.09)	**<0.001**
EQ-5D index score[4]	0.82$_{a}$ (0.10)	0.76$_{b}$ (0.13)	0.72$_{c}$ (0.14)	0.64$_{d}$ (0.18)	**<0.001**

	Depression severity (PHQ-9 score)				
	Minimal (Score 0-4) n=150	Mild (Score 5-9) n=368	Moderate (Score 10-14) n=337	Moderately Severe/Severe (Score 15-27) n=376	Omnibus *p*
EQ VAS score	76.72_a (21.15)	67.04_b (23.55)	65.95_b (20.98)	58.06_c (24.16)	**<0.001**
WPAI, mean (SD)					
Absenteeism (%)	9.45_a (23.54)	$12.07_{a,b}$ (23.64)	$17.17_{a,b}$ (27.59)	18.75_b (29.44)	**0.012**
Presenteeism (%)	18.89_a (22.30)	30.00_b (25.41)	38.57_c (25.99)	42.64_c (26.84)	**<0.001**
Total work productivity impairment (%)	25.21_a (29.68)	36.30_b (30.36)	46.59_c (30.85)	50.42_c (30.95)	**<0.001**
Activity impairment (%)	24.73_a (24.10)	34.86_b (27.01)	42.82_c (27.04)	49.36_d (28.14)	**<0.001**
HCRU in past 6 months, mean (SD)					
Healthcare provider visits	4.29_a (5.43)	4.60_a (7.02)	$5.64_{a,b}$ (7.89)	6.97_b (9.50)	**<0.001**
Psychiatrist visits	$0.47_{a,b}$ (1.43)	0.30_a (1.05)	$0.66_{a,b}$ (3.66)	0.79_b (2.14)	**0.031**
Psychologist/therapist visits	0.85_a (3.28)	$1.16_{a,b}$ (4.55)	$1.41_{a,b}$ (4.59)	2.05_b (5.02)	**0.015**
ER visits	0.20_a (0.49)	0.28_a (0.75)	$0.50_{a,b}$ (2.08)	0.62_b (1.50)	**0.002**
Hospitalizations	0.06_a (0.26)	0.30_a (2.29)	0.57_a (4.99)	0.34_a (1.07)	0.337
Annualized indirect costs ($), mean (SD)					
Absenteeism-related costs	$2,864_a$ (8,111)	$3,941_a$ (8,859)	$6,110_{a,b}$ (12,610)	$7,111_b$ (15,710)	**0.008**
Presenteeism-related costs	$6,772_a$ (10,159)	$9,428_{a,b}$ (11,397)	$11,025_b$ (11,372)	$11,062_{b,c}$ (11,440)	**0.009**
Total indirect costs	$9,636_a$ (14,444)	$13,369_{a,b}$ (15,184)	$17,135_{b,c}$ (17,017)	$18,173_c$ (19,333)	**<0.001**
Annualized direct medical costs ($), mean (SD)					
HCP visits costs	$2,706_a$ (3,458)	$2,868_a$ (4,332)	$3,480_{a,b}$ (4,933)	$4,372_b$ (5,936)	**<0.001**
ER visits costs	469_a (1,152)	646_a (1,744)	$1,153_{a,b}$ (4,820)	$1,450_b$ (3,500)	**0.002**
Hospitalizations costs	$1,784_a$ (7,840)	$8,748_a$ (64,412)	$16,219_a$ (138,868)	$9,893_a$ (30,870)	0.326
Total direct medical costs ($)	$4,959_a$ (9,685)	$12,263_a$ (65,759)	$20,851_a$ (139,323)	$15,716_a$ (33,607)	0.237

Note: Values in the same row and subtable not sharing the same subscript are significantly different at $p < .05$ in the two-sided test of equality for column proportions/means. Tests assume equal variances.

ER, emergency room; GAD-7, 7-item general anxiety disorder scale; HCRU, healthcare resource use; HRQoL, health-related quality of life; MCS, mental component summary; PCS, physical component summary; PHQ-9, 9-item Patient Health Questionnaire; WPAI, work productivity and activity impairment

Table A4. Sample characteristics, HRQoL, Work Productivity and Activity Impairment (WPAI), HCRU, and costs by depression severity among Asian respondents with self-reported physician-diagnosed depression

	**Depression severity (PHQ-9 score)**				
	**Minimal (Score 0-4)** **n=41**	**Mild (Score 5-9)** **n=102**	**Moderate (Score 10-14)** **n=64**	**Moderately Severe/Severe (Score 15-27)** **n=75**	**Omnibus *p***
Female, n (%)	27 (65.9)$_a$	73 (71.6)$_a$	41 (64.1)$_a$	47 (62.7)$_a$	0.604
Age, years, mean (SD)	46.27$_a$ (14.65)	37.75$_b$ (14.94)	34.34$_b$ (14.48)	34.87$_b$ (14.17)	**<0.001**
Married/living with partner, n (%)	21 (51.2)$_a$	45 (44.1)$_{a,b}$	23 (35.9)$_{a,b}$	20 (26.7)$_b$	**0.033**
University degree or higher, n (%)	29 (70.7)$_a$	62 (60.8)$_a$	33 (51.6)$_a$	43 (57.3)$_a$	0.435
Employed, n (%)	30 (73.2	72 (70.6)$_a$	37 (57.8)$_a$	45 (60.0)$_a$	0.180
Household income, n (%)					0.427
<$25,000	5 (12.2)$_a$	18 (17.6)$_a$	14 (21.9)$_a$	16 (21.3)$_a$	
$25,000 to <$50,000	3 (7.3)$_a$	15 (14.7)$_a$	7 (10.9)$_a$	15 (20.0)$_a$	
$50,000 to <$100,000	13 (31.7)$_a$	35 (34.3)$_a$	20 (31.3)$_a$	20 (26.7)$_a$	
$100,000 +	18 (43.9)$_a$	30 (29.4)$_a$	17 (26.6)$_a$	18 (24.0)$_a$	
Decline to answer	2 (4.9)$_a$	4 (3.9)$_a$	6 (9.4)$_a$	6 (8.0)$_a$	
Health insurance, n (%)					0.165
Commercially insured	34 (82.9)$_a$	72 (70.6)$_{a,b}$	37 (57.8)$_b$	45 (60.0)$_{a,b}$	
Medicaid	1 (2.4)$_a$	10 (9.8)$_a$	5 (7.8)$_a$	6 (8.0)$_a$	
Medicare	5 (12.2)$_a$	9 (8.8)$_a$	9 (14.1)$_a$	7 (9.3)$_a$	
Other type of insurance	1 (2.4)$_a$	4 (3.9)$_a$	4 (6.3)$_a$	6 (8.0)$_a$	
Not insured	0 (0.0)$_a$	7 (6.9)$_a$	9 (14.1)$_a$	11 (14.7)$_a$	
CCI score, mean (SD)	0.20$_a$ (0.56)	0.22$_a$ (0.61)	0.08$_a$ (0.37)	0.08$_a$ (0.32)	0.165
Days exercising, mean (SD)	10.78$_a$ (9.62)	8.61$_a$ (9.66)	8.06$_a$ (8.71)	7.41$_a$ (9.13)	0.305
Current prescription use for depression, yes, n (%)	25 (61.0)$_a$	49 (48.0)$_a$	34 (53.1)$_a$	41 (54.7)$_a$	0.544
PHQ-9 score, mean (SD)	2.27$_a$ (1.48)	6.95$_b$ (1.37)	11.88$_c$ (1.39)	19.17$_d$ (3.74)	**<0.001**
HRQoL					
GAD-7 score, mean (SD)	2.41$_a$ (1.92)	6.52$_b$ (3.95)	8.61$_c$ (3.75)	12.39$_d$ (5.56)	**<0.001**
MCS score, mean (SD)	47.01$_a$ (8.01)	37.77$_b$ (8.16)	34.71$_b$ (7.36)	26.96$_c$ (9.85)	**<0.001**
PCS score, mean (SD)	52.90$_a$ (7.40)	53.12$_a$ (8.92)	51.70$_a$ (9.19)	50.91$_a$ (9.53)	0.381
SF-6D utility score[3]	0.72$_a$ (0.10)	0.64$_b$ (0.08)	0.62$_b$ (0.08)	0.57$_c$ (0.07)	**<0.001**
EQ-5D index score[4]	0.86$_a$ (0.11)	0.78$_b$ (0.10)	0.74$_b$ (0.13)	0.68$_c$ (0.15)	**<0.001**

	Depression severity (PHQ-9 score)				
	Minimal (Score 0-4) n=41	Mild (Score 5-9) n=102	Moderate (Score 10-14) n=64	Moderately Severe/Severe (Score 15-27) n=75	Omnibus *p*
EQ VAS score	74.98_{a} (18.24)	71.41_{a} (20.85)	$67.69_{a,b}$ (21.01)	62.20_{b} (21.52)	**0.005**
WPAI, mean (SD)					
Absenteeism (%)	4.25_{a} (15.94)	8.45_{a} (17.14)	8.06_{a} (17.76)	20.07_{b} (22.48)	**0.002**
Presenteeism (%)	12.86_{a} (18.43)	26.92_{a} (25.00)	28.79_{a} (23.55)	46.10_{b} (27.83)	**<0.001**
Total work productivity impairment (%)	14.25_{a} (21.78)	32.14_{b} (28.85)	$32.36_{a,b}$ (27.64)	53.34_{c} (30.98)	**<0.001**
Activity impairment (%)	14.15_{a} (15.49)	28.73_{b} (24.44)	$36.25_{b,c}$ (27.17)	46.93_{c} (27.99)	**<0.001**
HCRU in past 6 months, mean (SD)					
Healthcare provider visits	3.85_{a} (4.26)	4.64_{a} (5.57)	5.66_{a} (9.31)	6.80_{a} (10.74)	0.191
Psychiatrist visits	0.41_{a} (0.97)	0.45_{a} (1.32)	0.77_{a} (2.72)	0.71_{a} (1.60)	0.576
Psychologist/therapist visits	0.68_{a} (2.96)	1.13_{a} (3.78)	2.14_{a} (6.05)	3.37_{a} (9.18)	0.054
ER visits	0.12_{a} (0.40)	0.25_{a} (0.68)	0.16_{a} (0.41)	0.21_{a} (0.81)	0.690
Hospitalizations	0.00_{a} (0.00)	0.04_{a} (0.20)	0.08_{a} (0.37)	0.21_{a} (0.78)	**0.039**
Annualized indirect costs ($), mean (SD)					
Absenteeism-related costs	$2,162_{a}$ (8,797)	$3,059_{a}$ (6,766)	$4,214_{a}$ (11,406)	$7,064_{a}$ (9,298)	0.061
Presenteeism-related costs	$5,209_{a}$ (6,430)	$8,935_{a}$ (10,827)	$10,689_{a}$ (11,812)	$12,700_{a}$ (11,961	**0.027**
Total indirect costs	$7,370_{a}$ (11,761)	$11,951_{a}$ (14,415)	$14,903_{a}$ (21,784)	$19,764_{a}$ (17,579)	**0.011**
Annualized direct medical costs ($), mean (SD)					
HCP visits costs	$2,547_{a}$ (2,895)	$2,863_{a}$ (3,394)	$3,446_{a}$ (5,613)	$4,181_{a}$ (6,526)	0.235
ER visits costs	291_{a} (956)	564_{a} (1,582)	363_{a} (944)	485_{a} (1,867)	0.717
Hospitalizations costs	0.00_{a} (0.00)	$1,179_{a}$ (5,921)	$2,459_{a}$ (11,921)	$6,044_{a}$ (21,695)	**0.047**
Total direct medical costs ($)	$2,838_{a}$ (3,028)	$4,607_{a}$ (7,589)	$6,267_{a}$ (13,857)	$10,710_{a}$ (23,341)	**0.015**

Note: Values in the same row and subtable not sharing the same subscript are significantly different at $p < .05$ in the two-sided test of equality for column proportions/means. Tests assume equal variances.

ER, emergency room; GAD-7, 7-item general anxiety disorder scale; HCRU, healthcare resource use; HRQoL, health-related quality of life; MCS, mental component summary; PCS, physical component summary; PHQ-9, 9-item Patient Health Questionnaire; WPAI, work productivity and activity impairment

Table A5. Patient activation by depression severity for each race/ethnicity group among adults with self-reported physician-diagnosed depression

	Depression severity (PHQ-9 score)				
	**Minimal (score 0-4)**	**Mild (score 5-9)**	**Moderate (score 10-14)**	**Moderately Severe/Severe (score 15-27)**	**Omnibus *p***
**White respondents**	**n=1,241**	**n=1,981**	**n=1,392**	**n=1,350**	
PAM score, mean (SD)	64.76$_{a}$ (11.65)	62.07$_{b}$ (11.17)	60.47$_{c}$ (11.31)	58.22$_{d}$ (11.93)	**<0.001**
PAM level, n (%)					**<0.001**
Level 1 (Disengaged & overwhelmed)	49 (3.9)$_{a}$	119 (6.0)$_{a}$	144 (10.3)$_{b}$	233 (17.3)$_{c}$	
Level 2 (Becoming aware but still struggling	168 (13.5)$_{a}$	352 (17.8)$_{b}$	311 (22.3)$_{c}$	312 (23.1)$_{c}$	
Level 3 (Taking action & gaining control)	682 (55.0)$_{a,b}$	1,126 (56.8)$_{a}$	697 (50.1)$_{b,c}$	618 (45.8)$_{c}$	
Level 4 (Maintaining behaviors & pushing further)	342 (27.6)$_{a}$	384 (19.4)$_{b}$	240 (17.2)$_{b,c}$	187 (13.9)$_{c}$	
**Black/African-American respondents**	**n=132**	**n=219**	**n=177**	**n=211**	
PAM score, mean (SD)	64.23$_{a}$ (11.58)	63.89$_{a}$ (12.14)	58.61$_{b}$ (12.65)	60.81$_{a,b}$ (12.47)	**<0.001**
PAM level, n (%)					**<0.001**
Level 1 (Disengaged & overwhelmed)	5 (3.8)$_{a}$	11 (5.0)$_{a}$	35 (19.8)$_{b}$	27 (12.8)$_{b}$	
Level 2 (Becoming aware but still struggling	17 (12.9)$_{a}$	37 (16.9)$_{a}$	36 (20.3)$_{a}$	43 (20.4)$_{a}$	
Level 3 (Taking action & gaining control)	77 (58.3)$_{a}$	108 (49.3)$_{a}$	82 (46.3)$_{a}$	98 (46.4)$_{a}$	
Level 4 (Maintaining behaviors & pushing further)	33 (25.0)$_{a,b}$	63 (28.8)$_{a}$	24 (13.6)$_{b}$	43 (20.4)$_{a,b}$	
**Hispanic respondents**	**n=150**	**n=368**	**n=337**	**n=376**	
PAM score, mean (SD)	64.64$_{a}$ (12.88)	63.41$_{a}$ (12.50)	58.61$_{b}$ (12.63)	58.41$_{b}$ (12.47)	**<0.001**
PAM level, n (%)					**<0.001**
Level 1 (Disengaged & overwhelmed)	9 (6.0)$_{a}$	19 (5.2)$_{a}$	63 (18.7)$_{b}$	67 (17.8)$_{b}$	
Level 2 (Becoming aware but still struggling	19 (12.7)$_{a}$	67 (18.2)$_{a}$	76 (22.6)$_{a}$	78 (20.7)$_{a}$	
Level 3 (Taking action & gaining control)	75 (50.0)$_{a,b}$	193 (52.4)$_{a}$	143 (42.4)$_{b}$	174 (46.3)$_{a,b}$	
Level 4 (Maintaining behaviors & pushing further)	47 (31.3)$_{a}$	89 (24.2)$_{a,b}$	55 (16.3)$_{b,c}$	57 (15.2)$_{c}$	
**Asian respondents**	**n=41**	**n=102**	**n=64**	**n=75**	
PAM score, mean (SD)	64.26$_{a}$ (12.31)	60.86$_{a}$ (10.56)	58.94$_{a}$ (9.17)	59.21$_{a}$ (12.93)	0.076
PAM level, n (%)					0.213
Level 1 (Disengaged & overwhelmed)	2 (4.9)$_{a}$	7 (6.9)$_{a}$	6 (9.4)$_{a}$	14 (18.7)$_{a}$	
Level 2 (Becoming aware but still struggling	7 (17.1)$_{a}$	20 (19.6)$_{a}$	14 (21.9)$_{a}$	13 (17.3)$_{a}$	
Level 3 (Taking action & gaining control)	22 (53.7)$_{a}$	60 (58.8)$_{a}$	37 (57.8)$_{a}$	36 (48.0)$_{a}$	
Level 4 (Maintaining behaviors & pushing further)	10 (24.4)$_{a}$	15 (14.7)$_{a}$	7 (10.9)$_{a}$	12 (16.0)$_{a}$	

Note: Values in the same row and subtable not sharing the same subscript are significantly different at *p*< .05 in the two-sided test of equality for column proportions/means. Tests assume equal variances. PAM; patient activation measure.

Table A6. Sensitivity analysis: Association of depression severity and race/ethnicity with patient activation: parameter estimates and adjusted mean PAM scores among adults with self-reported physician-diagnosed depression

	**Parameter estimates**	
	**β (SE)**	**p-value**
**Depression severity (PHQ-9 score)**[1]	-0.306 (0.021)	**<0.001**
**Race/ethnicity**[2]		
White (reference)	0	
Black/African American	1.281 (0.455)	**0.005**
Hispanic	0.407 (0.368)	0.270
Asian	-0.375 (0.707)	0.596
	**Adjusted PAM scores**	
	**Mean**	**95% CI**
**Race/ethnicity**		
White (reference)	60.06	58.47 - 61.65
Black/African American	61.34	59.58 - 63.10
Hispanic	60.46	58.76 - 62.17
Asian	59.68	57.60 - 61.76

Note: continuous predictors were centered due to multicollinearity when including interaction term; controlling for age, sex, marital status, days exercising, CCI score, and current prescription use for depression.

[1]Interpretation: For each 1-point increase in PHQ-9 score, the PAM score changes by an average of <β>, keeping other predictors constant.

[2]Interpretation: PAM scores change by an average of <β> for <Black/African American, Hispanic, or Asian respondents> compared to White respondents, keeping all other predictors constant.

CI, confidence interval; PHQ-9, 9-item Patient Health Questionnaire; SE, standard error

Table A7. Predictors of patient activation within race/ethnicity groups among adults with self-reported physician-diagnosed depression - parameter estimates

	**White n=5,964**		**Black/African American n=739**		**Hispanic n=1,231**		**Asian n=282**	
	**β[1] (SE)**	***P***	**β[1] (SE)**	***P***	**β[1] (SE)**	***P***	**β[1] (SE)**	***P***
(Intercept)	36.843 (2.061)	<0.001	50.587 (5.818)	<0.001	42.263 (4.632)	<0.001	26.164 (9.148)	0.004
PHQ-9 score	-0.111 (0.038)	**0.003**	-0.073 (0.106)	0.493	-0.124 (0.085)	0.145	-0.085 (0.149)	0.565
Household income (ref: $25,000)								
$25,000 to <$50,000	0.351 (0.469)	0.454	1.457 (1.204)	0.226	0.280 (0.985)	0.777	3.225 (2.225)	0.147
$50,000 to <$100,000	0.505 (0.475)	0.287	-0.503 (1.348)	0.709	-0.160 (1.048)	0.878	1.596 (1.993)	0.423
≥$100,000	1.400 (0.557)	**0.012**	-1.077 (1.862)	0.563	-0.328 (1.333)	0.806	1.649 (2.129)	0.438
Decline to answer	-0.415 (0.831)	0.618	-5.491 (2.611)	**0.035**	-0.561 (1.911)	0.769	3.008 (2.861)	0.293
Sex (ref: Male)								
Sex: Female	1.696 (0.314)	**<0.001**	0.853 (1.025)	0.406	0.814 (0.750)	0.278	3.503 (1.325)	**0.008**
Marital status (ref: Married/living with partner)								
Not married/living with partner[2]	-0.285 (0.314)	0.363	-0.533 (1.049)	0.611	-0.245 (0.744)	0.741	-0.935 (1.490)	0.530
Decline to answer	-1.726 (2.776)	0.534	-9.228 (6.061)	0.128	14.996 (9.402)	0.111	0c (0.000)	0.000
Education (ref: Less than university degree)								
University degree or higher	-0.034 (0.321)	0.914	0.254 (1.017)	0.803	0.011 (0.781)	0.989	0.469 (1.371)	0.732
Decline to Answer	-0.977 (3.610)	0.787	-16.014 (11.904)	0.179	-4.779 (7.706)	0.535	4.847 (10.502)	0.644
Insurance status (ref: Commercial)								
Medicaid	0.229 (0.504)	0.650	-2.229 (1.403)	0.112	-0.431 (1.097)	0.694	1.697 (2.410)	0.481
Medicare	-0.481 (0.452)	0.287	-1.532 (1.311)	0.243	-0.996 (1.148)	0.386	-2.135 (2.220)	0.336
Other type of insurance	0.001 (0.733)	0.999	-4.000 (2.065)	0.053	-5.690 (1.483)	**<0.001**	-0.197 (2.839)	0.945
Not insured	0.192 (0.553)	0.728	-4.734 (1.555)	**0.002**	-2.723 (1.058)	**0.010**	-3.987 (2.311)	0.084
Current prescription for depression (ref: Yes)	0		0		0		0	
No	-0.717 (0.307)	**0.019**	0.418 (0.931)	0.653	-1.049 (0.705)	0.137	-2.040 (1.305)	0.118
Age, years	0.071 (0.011)	**<0.001**	0.157 (0.035)	**<0.001**	0.136 (0.028)	**<0.001**	0.026 (0.051)	0.609
CCI score	0.548 (0.157)	**<0.001**	0.040 (0.510)	0.937	0.015 (0.421)	0.972	1.134 (1.292)	0.380
Days exercising in past month	0.169 (0.018)	**<0.001**	0.156 (0.059)	**0.008**	0.097 (0.043)	**0.023**	0.055 (0.069)	0.427
Number of HCP visits in past 6 months	0.049 (0.021)	**0.017**	-0.017 (0.070)	0.807	0.147 (0.046)	**0.001**	-0.247 (0.083)	**0.003**
Number of ER visits in past 6 months	0.149 (0.205)	0.465	0.141 (0.445)	0.752	-0.308 (0.246)	0.211	0.860 (1.080)	0.426
Number of hospitalizations in past 6 months	0.346 (0.290)	0.233	-0.084 (0.611)	0.891	-0.067 (0.118)	0.575	-0.304 (1.470)	0.836
GAD-7 score	0.104 (0.040)	**0.009**	0.060 (0.120)	0.616	0.085 (0.094)	0.368	0.371 (0.159)	**0.019**
Activity impairment (%)	0.017 (0.008)	**0.035**	-0.011 (0.022)	0.606	-0.040 (0.017)	**0.017**	0.071 (0.032)	**0.027**
MCS score	0.201 (0.021)	**<0.001**	0.021 (0.059)	0.725	0.163 (0.048)	**0.001**	0.370 (0.088)	**<0.001**
PCS score	0.217 (0.021)	**<0.001**	0.104 (0.062)	0.096	0.199 (0.049)	**<0.001**	0.270 (0.099)	**0.006**

Note: Yellow highlights denote strongest predictors for that race/ethnicity group.

[1]Interpretation: continuous predictors - For each 1-unit increase in predictor, PAM score changes by an average of <β>, keeping other predictors constant; categorical predictors - PAM scores change by an average of <β> for <category> compared to reference group, keeping all other predictors constant.

[2]Includes single, not married, separated, divorced, widowed

CCI, Charlson Comorbidity Index; ER, emergency room; GAD-7, 7-item general anxiety disorder scale; HCP, healthcare provider; MCS, mental component summary; PCS, physical component summary; PHQ-9, 9-item Patient Health Questionnaire; ref, reference; SE, standard error
